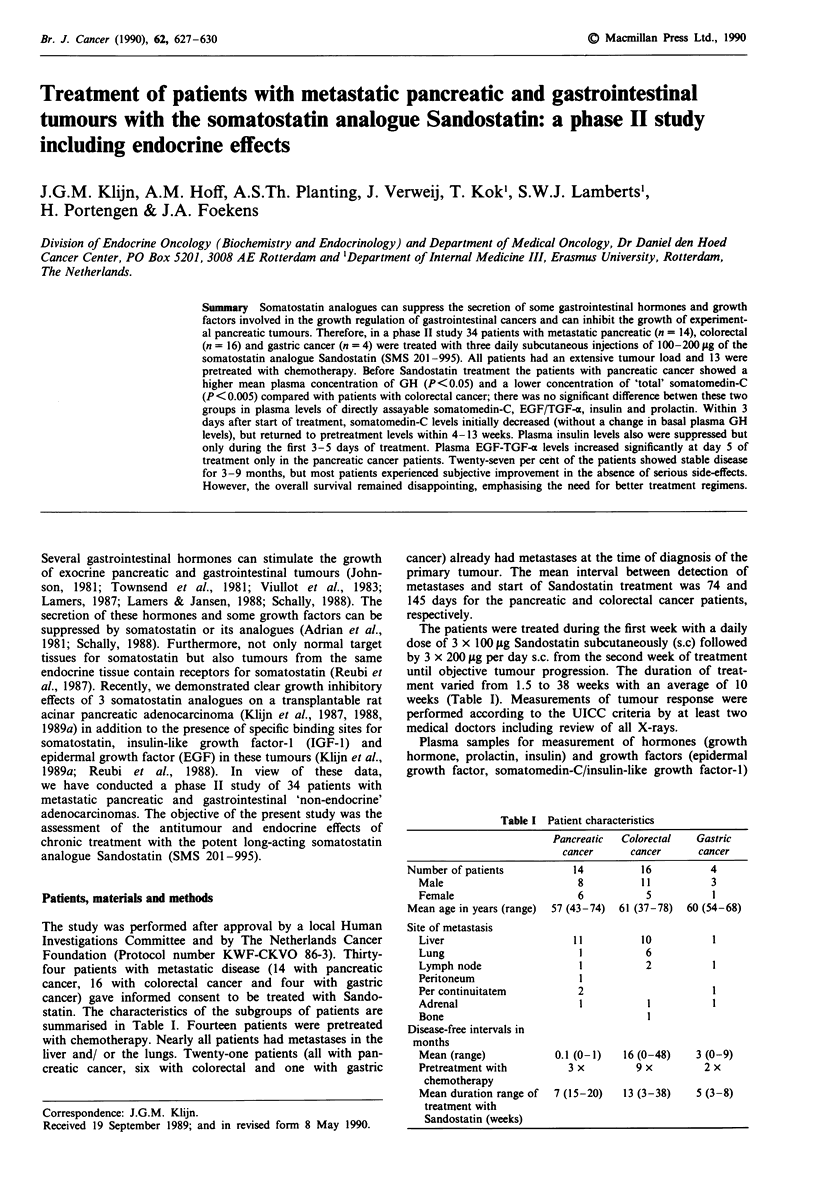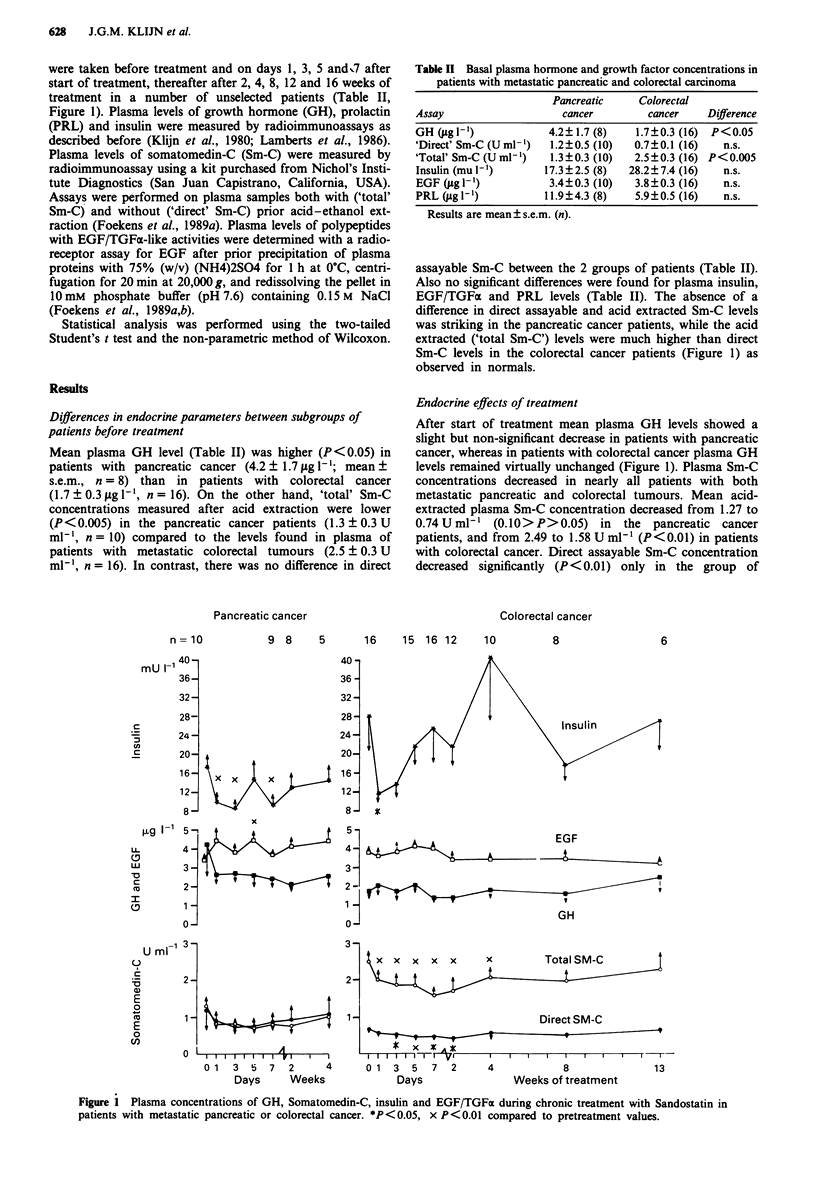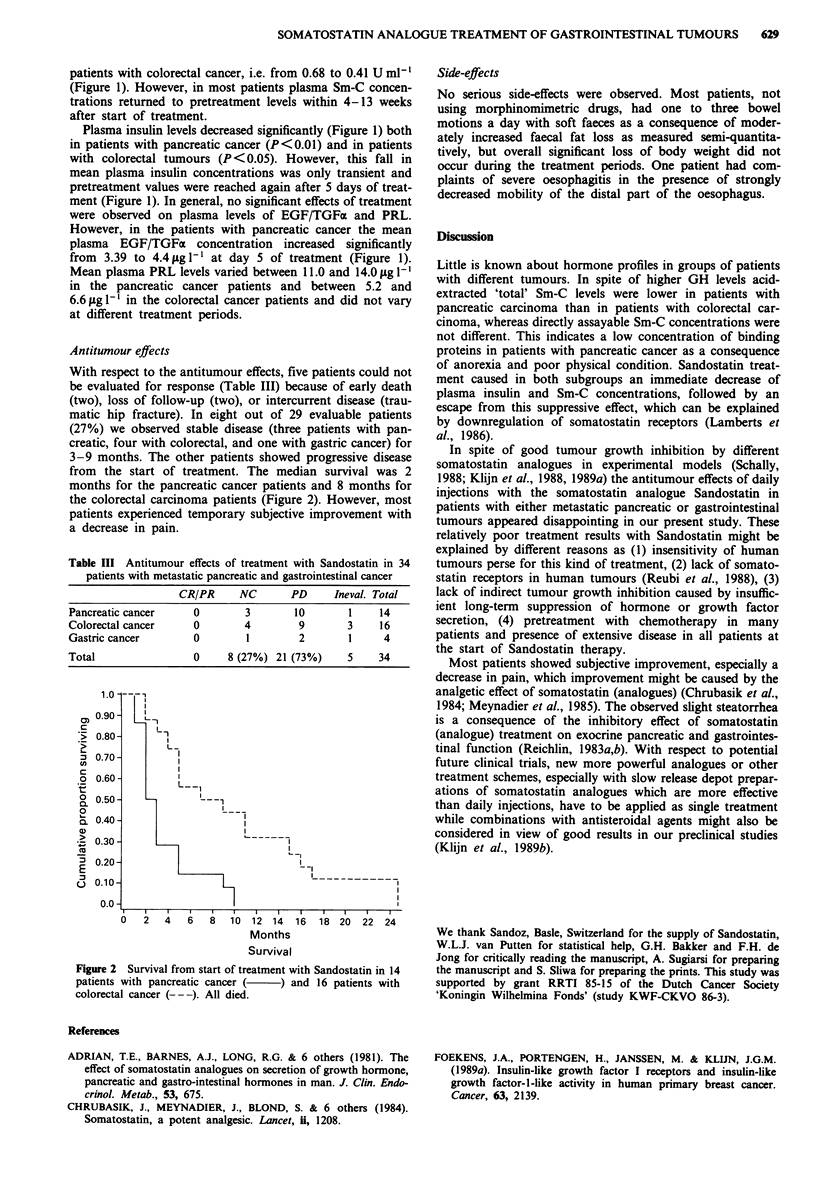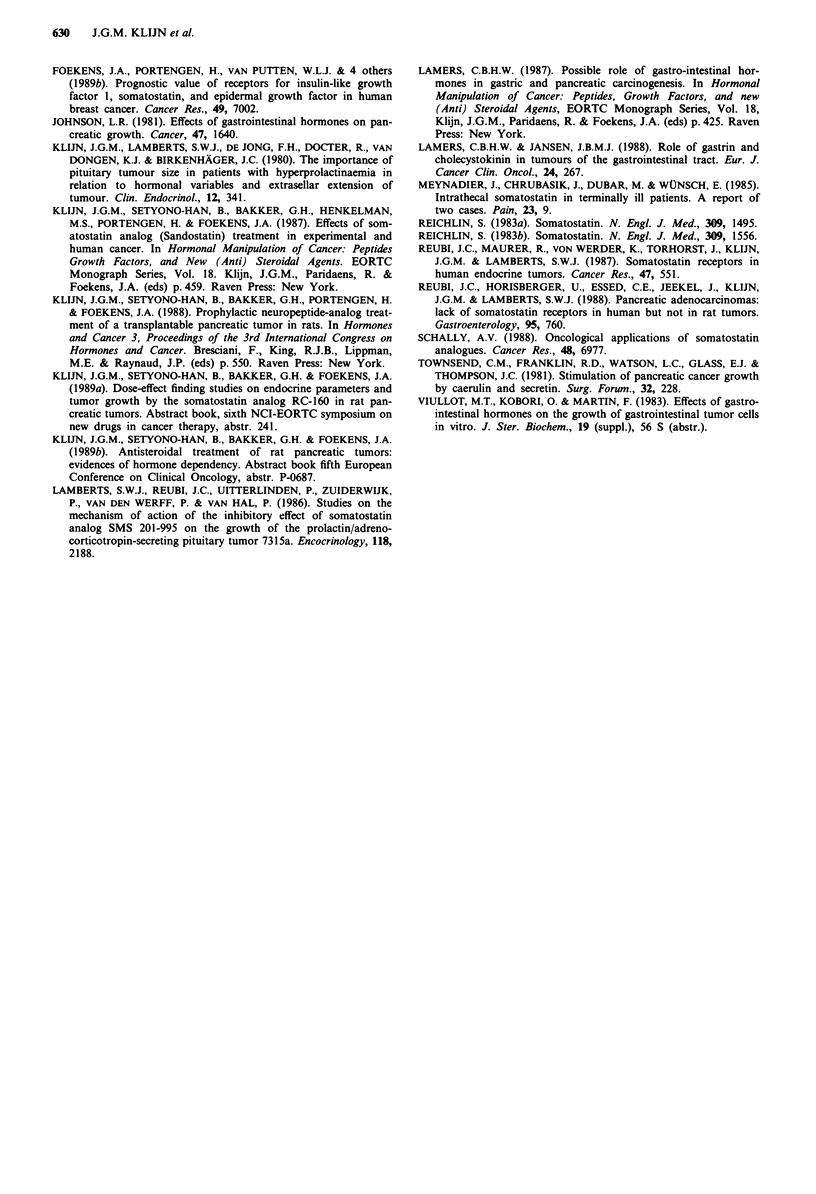# Treatment of patients with metastatic pancreatic and gastrointestinal tumours with the somatostatin analogue Sandostatin: a phase II study including endocrine effects.

**DOI:** 10.1038/bjc.1990.343

**Published:** 1990-10

**Authors:** J. G. Klijn, A. M. Hoff, A. S. Planting, J. Verweij, T. Kok, S. W. Lamberts, H. Portengen, J. A. Foekens

**Affiliations:** Division of Endocrine Oncology (Biochemistry and Endocrinology), Dr Daniel den Hoed Cancer Center, Rotterdam, The Netherlands.

## Abstract

Somatostatin analogues can suppress the secretion of some gastrointestinal hormones and growth factors involved in the growth regulation of gastrointestinal cancers and can inhibit the growth of experimental pancreatic tumours. Therefore, in a phase II study 34 patients with metastatic pancreatic (n = 14), colorectal (n = 16) and gastric cancer (n = 4) were treated with three daily subcutaneous injections of 100-200 micrograms of the somatostatin analogue Sandostatin (SMS 201-995). All patients had an extensive tumour load and 13 were pretreated with chemotherapy. Before Sandostatin treatment the patients with pancreatic cancer showed a higher mean plasma concentration of GH (P less than 0.05) and a lower concentration of 'total' somatomedin-C (P less than 0.005) compared with patients with colorectal cancer; there was no significant difference between these two groups in plasma levels of directly assayable somatomedin-C, EGF/TGF-alpha, insulin and prolactin. Within 3 days after start of treatment, somatomedin-C levels initially decreased (without a change in basal plasma GH levels), but returned to pretreatment levels within 4-13 weeks. Plasma insulin levels also were suppressed but only during the first 3-5 days of treatment. Plasma EGF-TGF-alpha levels increased significantly at day 5 of treatment only in the pancreatic cancer patients. Twenty-seven per cent of the patients showed stable disease for 3-9 months, but most patients experienced subjective improvement in the absence of serious side-effects. However, the overall survival remained disappointing, emphasising the need for better treatment regimens.


*Br. J. Cancer* (1990), **62**, 627–630 © Macmillan Press Ltd., 1990

### Treatment of patients with metastatic pancreatic and gastrointestinal tumours with the somatostatin analogue Sandostatin: a phase II study including endocrine effects

J.G.M. Klijn, A.M. Hoff, A.S.Th. Planting, J. Verweij, T. Kok[1], S.W.J. Lamberts[1], H. Portengen & J.A. Foekens

*Division of Endocrine Oncology (Biochemistry and Endocrinology) and Department of Medical Oncology, Dr Daniel den Hoed Cancer Center, PO Box 5201, 3008 AE Rotterdam and [1]Department of Internal Medicine III, Erasmus University, Rotterdam, The Netherlands.*

**Summary** Somatostatin analogues can suppress the secretion of some gastrointestinal hormones and growth factors involved in the growth regulation of gastrointestinal cancers and can inhibit the growth of experimental pancreatic tumours. Therefore, in a phase II study 34 patients with metastatic pancreatic ($n = 14$), colorectal ($n = 16$) and gastric cancer ($n = 4$) were treated with three daily subcutaneous injections of 100–200 μg of the somatostatin analogue Sandostatin (SMS 201–995). All patients had an extensive tumour load and 13 were pretreated with chemotherapy. Before Sandostatin treatment the patients with pancreatic cancer showed a higher mean plasma concentration of GH ($P<0.05$) and a lower concentration of 'total' somatomedin-C ($P<0.005$) compared with patients with colorectal cancer; there was no significant difference betwen these two groups in plasma levels of directly assayable somatomedin-C, EGF/TGF-α, insulin and prolactin. Within 3 days after start of treatment, somatomedin-C levels initially decreased (without a change in basal plasma GH levels), but returned to pretreatment levels within 4–13 weeks. Plasma insulin levels also were suppressed but only during the first 3–5 days of treatment. Plasma EGF-TGF-α levels increased significantly at day 5 of treatment only in the pancreatic cancer patients. Twenty-seven per cent of the patients showed stable disease for 3–9 months, but most patients experienced subjective improvement in the absence of serious side-effects. However, the overall survival remained disappointing, emphasising the need for better treatment regimens.

Several gastrointestinal hormones can stimulate the growth of exocrine pancreatic and gastrointestinal tumours (Johnson, 1981; Townsend *et al.*, 1981; Viullot *et al.*, 1983; Lamers, 1987; Lamers & Jansen, 1988; Schally, 1988). The secretion of these hormones and some growth factors can be suppressed by somatostatin or its analogues (Adrian *et al.*, 1981; Schally, 1988). Furthermore, not only normal target tissues for somatostatin but also tumours from the same endocrine tissue contain receptors for somatostatin (Reubi *et al.*, 1987). Recently, we demonstrated clear growth inhibitory effects of 3 somatostatin analogues on a transplantable rat acinar pancreatic adenocarcinoma (Klijn *et al.*, 1987, 1988, 1989*a*) in addition to the presence of specific binding sites for somatostatin, insulin-like growth factor-1 (IGF-1) and epidermal growth factor (EGF) in these tumours (Klijn *et al.*, 1989*a*; Reubi *et al.*, 1988). In view of these data, we have conducted a phase II study of 34 patients with metastatic pancreatic and gastrointestinal 'non-endocrine' adenocarcinomas. The objective of the present study was the assessment of the antitumour and endocrine effects of chronic treatment with the potent long-acting somatostatin analogue Sandostatin (SMS 201–995).

### Patients, materials and methods

The study was performed after approval by a local Human Investigations Committee and by The Netherlands Cancer Foundation (Protocol number KWF-CKVO 86-3). Thirty-four patients with metastatic disease (14 with pancreatic cancer, 16 with colorectal cancer and four with gastric cancer) gave informed consent to be treated with Sandostatin. The characteristics of the subgroups of patients are summarised in Table I. Fourteen patients were pretreated with chemotherapy. Nearly all patients had metastases in the liver and/ or the lungs. Twenty-one patients (all with pancreatic cancer, six with colorectal and one with gastric cancer) already had metastases at the time of diagnosis of the primary tumour. The mean interval between detection of metastases and start of Sandostatin treatment was 74 and 145 days for the pancreatic and colorectal cancer patients, respectively.

The patients were treated during the first week with a daily dose of 3 × 100 μg Sandostatin subcutaneously (s.c) followed by 3 × 200 μg per day s.c. from the second week of treatment until objective tumour progression. The duration of treatment varied from 1.5 to 38 weeks with an average of 10 weeks (Table I). Measurements of tumour response were performed according to the UICC criteria by at least two medical doctors including review of all X-rays.

Plasma samples for measurement of hormones (growth hormone, prolactin, insulin) and growth factors (epidermal growth factor, somatomedin-C/insulin-like growth factor-1)

**Table I** Patient characteristics

| | *Pancreatic cancer* | *Colorectal cancer* | *Gastric cancer* |
|---|---|---|---|
| Number of patients | 14 | 16 | 4 |
| Male | 8 | 11 | 3 |
| Female | 6 | 5 | 1 |
| Mean age in years (range) | 57 (43–74) | 61 (37–78) | 60 (54–68) |
| Site of metastasis | | | |
| Liver | 11 | 10 | 1 |
| Lung | 1 | 6 | |
| Lymph node | 1 | 2 | 1 |
| Peritoneum | 1 | | |
| Per continuitatem | 2 | | 1 |
| Adrenal | 1 | 1 | 1 |
| Bone | | 1 | |
| Disease-free intervals in months | | | |
| Mean (range) | 0.1 (0–1) | 16 (0–48) | 3 (0–9) |
| Pretreatment with chemotherapy | 3 × | 9 × | 2 × |
| Mean duration range of treatment with Sandostatin (weeks) | 7 (15–20) | 13 (3–38) | 5 (3–8) |

Correspondence: J.G.M. Klijn.
Received 19 September 1989; and in revised form 8 May 1990.

were taken before treatment and on days 1, 3, 5 and 7 after start of treatment, thereafter after 2, 4, 8, 12 and 16 weeks of treatment in a number of unselected patients (Table II, Figure 1). Plasma levels of growth hormone (GH), prolactin (PRL) and insulin were measured by radioimmunoassays as described before (Klijn *et al.*, 1980; Lamberts *et al.*, 1986). Plasma levels of somatomedin-C (Sm-C) were measured by radioimmunoassay using a kit purchased from Nichol's Institute Diagnostics (San Juan Capistrano, California, USA). Assays were performed on plasma samples both with ('total' Sm-C) and without ('direct' Sm-C) prior acid–ethanol extraction (Foekens *et al.*, 1989*a*). Plasma levels of polypeptides with EGF/TGFα-like activities were determined with a radioreceptor assay for EGF after prior precipitation of plasma proteins with 75% (w/v) (NH4)2SO4 for 1 h at 0°C, centrifugation for 20 min at 20,000 *g*, and redissolving the pellet in 10 mM phosphate buffer (pH 7.6) containing 0.15 M NaCl (Foekens *et al.*, 1989*a*,*b*).

Statistical analysis was performed using the two-tailed Student's *t* test and the non-parametric method of Wilcoxon.

### Results

### *Differences in endocrine parameters between subgroups of patients before treatment*

Mean plasma GH level (Table II) was higher ($P<0.05$) in patients with pancreatic cancer ($4.2 \pm 1.7\ \mu g\ l^{-1}$; mean ± s.e.m., $n = 8$) than in patients with colorectal cancer ($1.7 \pm 0.3\ \mu g\ l^{-1}$, $n = 16$). On the other hand, 'total' Sm-C concentrations measured after acid extraction were lower ($P<0.005$) in the pancreatic cancer patients ($1.3 \pm 0.3$ U $ml^{-1}$, $n = 10$) compared to the levels found in plasma of patients with metastatic colorectal tumours ($2.5 \pm 0.3$ U $ml^{-1}$, $n = 16$). In contrast, there was no difference in direct

**Table II** Basal plasma hormone and growth factor concentrations in patients with metastatic pancreatic and colorectal carcinoma

| *Assay* | *Pancreatic cancer* | *Colorectal cancer* | *Difference* |
|---|---|---|---|
| GH ($\mu g\ l^{-1}$) | 4.2±1.7 (8) | 1.7±0.3 (16) | $P<0.05$ |
| 'Direct' Sm-C (U $ml^{-1}$) | 1.2±0.5 (10) | 0.7±0.1 (16) | n.s. |
| 'Total' Sm-C (U $ml^{-1}$) | 1.3±0.3 (10) | 2.5±0.3 (16) | $P<0.005$ |
| Insulin (mu $l^{-1}$) | 17.3±2.5 (8) | 28.2±7.4 (16) | n.s. |
| EGF ($\mu g\ l^{-1}$) | 3.4±0.3 (10) | 3.8±0.3 (16) | n.s. |
| PRL ($\mu g\ l^{-1}$) | 11.9±4.3 (8) | 5.9±0.5 (16) | n.s. |

Results are mean ± s.e.m. (*n*).

assayable Sm-C between the 2 groups of patients (Table II). Also no significant differences were found for plasma insulin, EGF/TGFα and PRL levels (Table II). The absence of a difference in direct assayable and acid extracted Sm-C levels was striking in the pancreatic cancer patients, while the acid extracted ('total Sm-C') levels were much higher than direct Sm-C levels in the colorectal cancer patients (Figure 1) as observed in normals.

### *Endocrine effects of treatment*

After start of treatment mean plasma GH levels showed a slight but non-significant decrease in patients with pancreatic cancer, whereas in patients with colorectal cancer plasma GH levels remained virtually unchanged (Figure 1). Plasma Sm-C concentrations decreased in nearly all patients with both metastatic pancreatic and colorectal tumours. Mean acid-extracted plasma Sm-C concentration decreased from 1.27 to 0.74 U $ml^{-1}$ ($0.10>P>0.05$) in the pancreatic cancer patients, and from 2.49 to 1.58 U $ml^{-1}$ ($P<0.01$) in patients with colorectal cancer. Direct assayable Sm-C concentration decreased significantly ($P<0.01$) only in the group of

**Figure 1** Plasma concentrations of GH, Somatomedin-C, insulin and EGF/TGFα during chronic treatment with Sandostatin in patients with metastatic pancreatic or colorectal cancer. *$P<0.05$, × $P<0.01$ compared to pretreatment values.

patients with colorectal cancer, i.e. from 0.68 to 0.41 U $ml^{-1}$ (Figure 1). However, in most patients plasma Sm-C concentrations returned to pretreatment levels within 4–13 weeks after start of treatment.

Plasma insulin levels decreased significantly (Figure 1) both in patients with pancreatic cancer ($P<0.01$) and in patients with colorectal tumours ($P<0.05$). However, this fall in mean plasma insulin concentrations was only transient and pretreatment values were reached again after 5 days of treatment (Figure 1). In general, no significant effects of treatment were observed on plasma levels of EGF/TGFα and PRL. However, in the patients with pancreatic cancer the mean plasma EGF/TGFα concentration increased significantly from 3.39 to 4.4 $\mu g\ l^{-1}$ at day 5 of treatment (Figure 1). Mean plasma PRL levels varied between 11.0 and 14.0 $\mu g\ l^{-1}$ in the pancreatic cancer patients and between 5.2 and 6.6 $\mu g\ l^{-1}$ in the colorectal cancer patients and did not vary at different treatment periods.

### *Antitumour effects*

With respect to the antitumour effects, five patients could not be evaluated for response (Table III) because of early death (two), loss of follow-up (two), or intercurrent disease (traumatic hip fracture). In eight out of 29 evaluable patients (27%) we observed stable disease (three patients with pancreatic, four with colorectal, and one with gastric cancer) for 3–9 months. The other patients showed progressive disease from the start of treatment. The median survival was 2 months for the pancreatic cancer patients and 8 months for the colorectal carcinoma patients (Figure 2). However, most patients experienced temporary subjective improvement with a decrease in pain.

**Table III** Antitumour effects of treatment with Sandostatin in 34 patients with metastatic pancreatic and gastrointestinal cancer

| | *CR/PR* | *NC* | *PD* | *Ineval.* | *Total* |
|---|---|---|---|---|---|
| Pancreatic cancer | 0 | 3 | 10 | 1 | 14 |
| Colorectal cancer | 0 | 4 | 9 | 3 | 16 |
| Gastric cancer | 0 | 1 | 2 | 1 | 4 |
| Total | 0 | 8 (27%) | 21 (73%) | 5 | 34 |

**Figure 2** Survival from start of treatment with Sandostatin in 14 patients with pancreatic cancer (———) and 16 patients with colorectal cancer (– – –). All died.

### *Side-effects*

No serious side-effects were observed. Most patients, not using morphinomimetric drugs, had one to three bowel motions a day with soft faeces as a consequence of moderately increased faecal fat loss as measured semi-quantitatively, but overall significant loss of body weight did not occur during the treatment periods. One patient had complaints of severe oesophagitis in the presence of strongly decreased mobility of the distal part of the oesophagus.

### Discussion

Little is known about hormone profiles in groups of patients with different tumours. In spite of higher GH levels acid-extracted 'total' Sm-C levels were lower in patients with pancreatic carcinoma than in patients with colorectal carcinoma, whereas directly assayable Sm-C concentrations were not different. This indicates a low concentration of binding proteins in patients with pancreatic cancer as a consequence of anorexia and poor physical condition. Sandostatin treatment caused in both subgroups an immediate decrease of plasma insulin and Sm-C concentrations, followed by an escape from this suppressive effect, which can be explained by downregulation of somatostatin receptors (Lamberts *et al.*, 1986).

In spite of good tumour growth inhibition by different somatostatin analogues in experimental models (Schally, 1988; Klijn *et al.*, 1988, 1989*a*) the antitumour effects of daily injections with the somatostatin analogue Sandostatin in patients with either metastatic pancreatic or gastrointestinal tumours appeared disappointing in our present study. These relatively poor treatment results with Sandostatin might be explained by different reasons as (1) insensitivity of human tumours perse for this kind of treatment, (2) lack of somatostatin receptors in human tumours (Reubi *et al.*, 1988), (3) lack of indirect tumour growth inhibition caused by insufficient long-term suppression of hormone or growth factor secretion, (4) pretreatment with chemotherapy in many patients and presence of extensive disease in all patients at the start of Sandostatin therapy.

Most patients showed subjective improvement, especially a decrease in pain, which improvement might be caused by the analgetic effect of somatostatin (analogues) (Chrubasik *et al.*, 1984; Meynadier *et al.*, 1985). The observed slight steatorrhea is a consequence of the inhibitory effect of somatostatin (analogue) treatment on exocrine pancreatic and gastrointestinal function (Reichlin, 1983*a*,*b*). With respect to potential future clinical trials, new more powerful analogues or other treatment schemes, especially with slow release depot preparations of somatostatin analogues which are more effective than daily injections, have to be applied as single treatment while combinations with antisteroidal agents might also be considered in view of good results in our preclinical studies (Klijn *et al.*, 1989*b*).

We thank Sandoz, Basle, Switzerland for the supply of Sandostatin, W.L.J. van Putten for statistical help, G.H. Bakker and F.H. de Jong for critically reading the manuscript, A. Sugiarsi for preparing the manuscript and S. Sliwa for preparing the prints. This study was supported by grant RRTI 85-15 of the Dutch Cancer Society 'Koningin Wilhelmina Fonds' (study KWF-CKVO 86-3).

### References

ADRIAN, T.E., BARNES, A.J., LONG, R.G. & 6 others (1981). The effect of somatostatin analogues on secretion of growth hormone, pancreatic and gastro-intestinal hormones in man. *J. Clin. Endocrinol. Metab.*, **53**, 675.

CHRUBASIK, J., MEYNADIER, J., BLOND, S. & 6 others (1984). Somatostatin, a potent analgesic. *Lancet*, **ii**, 1208.

FOEKENS, J.A., PORTENGEN, H., JANSSEN, M. & KLIJN, J.G.M. (1989*a*). Insulin-like growth factor I receptors and insulin-like growth factor-1-like activity in human primary breast cancer. *Cancer*, **63**, 2139.

FOEKENS, J.A., PORTENGEN, H., VAN PUTTEN, W.L.J. & 4 others (1989*b*). Prognostic value of receptors for insulin-like growth factor 1, somatostatin, and epidermal growth factor in human breast cancer. *Cancer Res.*, **49**, 7002.

JOHNSON, L.R. (1981). Effects of gastrointestinal hormones on pancreatic growth. *Cancer*, **47**, 1640.

KLIJN, J.G.M., LAMBERTS, S.W.J., DE JONG, F.H., DOCTER, R., VAN DONGEN, K.J. & BIRKENHÄGER, J.C. (1980). The importance of pituitary tumour size in patients with hyperprolactinaemia in relation to hormonal variables and extrasellar extension of tumour. *Clin. Endocrinol.*, **12**, 341.

KLIJN, J.G.M., SETYONO-HAN, B., BAKKER, G.H., HENKELMAN, M.S., PORTENGEN, H. & FOEKENS, J.A. (1987). Effects of somatostatin analog (Sandostatin) treatment in experimental and human cancer. In *Hormonal Manipulation of Cancer: Peptides Growth Factors, and New (Anti) Steroidal Agents*. EORTC Monograph Series, Vol. 18. Klijn, J.G.M., Paridaens, R. & Foekens, J.A. (eds) p. 459. Raven Press: New York.

KLIJN, J.G.M., SETYONO-HAN, B., BAKKER, G.H., PORTENGEN, H. & FOEKENS, J.A. (1988). Prophylactic neuropeptide-analog treatment of a transplantable pancreatic tumor in rats. In *Hormones and Cancer 3, Proceedings of the 3rd International Congress on Hormones and Cancer*. Bresciani, F., King, R.J.B., Lippman, M.E. & Raynaud, J.P. (eds) p. 550. Raven Press: New York.

KLIJN, J.G.M., SETYONO-HAN, B., BAKKER, G.H. & FOEKENS, J.A. (1989*a*). Dose-effect finding studies on endocrine parameters and tumor growth by the somatostatin analog RC-160 in rat pancreatic tumors. Abstract book, sixth NCI-EORTC symposium on new drugs in cancer therapy, abstr. 241.

KLIJN, J.G.M., SETYONO-HAN, B., BAKKER, G.H. & FOEKENS, J.A. (1989*b*). Antisteroidal treatment of rat pancreatic tumors: evidences of hormone dependency. Abstract book fifth European Conference on Clinical Oncology, abstr. P-0687.

LAMBERTS, S.W.J., REUBI, J.C., UITTERLINDEN, P., ZUIDERWIJK, P., VAN DEN WERFF, P. & VAN HAL, P. (1986). Studies on the mechanism of action of the inhibitory effect of somatostatin analog SMS 201-995 on the growth of the prolactin/adrenocorticotropin-secreting pituitary tumor 7315a. *Encocrinology*, **118**, 2188.

LAMERS, C.B.H.W. (1987). Possible role of gastro-intestinal hormones in gastric and pancreatic carcinogenesis. In *Hormonal Manipulation of Cancer: Peptides, Growth Factors, and new (Anti) Steroidal Agents*, EORTC Monograph Series, Vol. 18, Klijn, J.G.M., Paridaens, R. & Foekens, J.A. (eds) p. 425. Raven Press: New York.

LAMERS, C.B.H.W. & JANSEN, J.B.M.J. (1988). Role of gastrin and cholecystokinin in tumours of the gastrointestinal tract. *Eur. J. Cancer Clin. Oncol.*, **24**, 267.

MEYNADIER, J., CHRUBASIK, J., DUBAR, M. & WÜNSCH, E. (1985). Intrathecal somatostatin in terminally ill patients. A report of two cases. *Pain*, **23**, 9.

REICHLIN, S. (1983*a*). Somatostatin. *N. Engl. J. Med.*, **309**, 1495.

REICHLIN, S. (1983*b*). Somatostatin. *N. Engl. J. Med.*, **309**, 1556.

REUBI, J.C., MAURER, R., VON WERDER, K., TORHORST, J., KLIJN, J.G.M. & LAMBERTS, S.W.J. (1987). Somatostatin receptors in human endocrine tumors. *Cancer Res.*, **47**, 551.

REUBI, J.C., HORISBERGER, U., ESSED, C.E., JEEKEL, J., KLIJN, J.G.M. & LAMBERTS, S.W.J. (1988). Pancreatic adenocarcinomas: lack of somatostatin receptors in human but not in rat tumors. *Gastroenterology*, **95**, 760.

SCHALLY, A.V. (1988). Oncological applications of somatostatin analogues. *Cancer Res.*, **48**, 6977.

TOWNSEND, C.M., FRANKLIN, R.D., WATSON, L.C., GLASS, E.J. & THOMPSON, J.C. (1981). Stimulation of pancreatic cancer growth by caerulin and secretin. *Surg. Forum.*, **32**, 228.

VIULLOT, M.T., KOBORI, O. & MARTIN, F. (1983). Effects of gastrointestinal hormones on the growth of gastrointestinal tumor cells in vitro. *J. Ster. Biochem.*, **19** (suppl.), 56 S (abstr.).